# 兔抗人胸腺细胞免疫球蛋白用于血液病患者第二次异基因造血干细胞移植的安全性

**DOI:** 10.3760/cma.j.issn.0253-2727.2022.10.009

**Published:** 2022-10

**Authors:** 阳 刘, 婷婷 韩, 瑶 陈, 欢 陈, 海霞 付, 圆圆 张, 峰蓉 王, 景枝 王, 晨华 闫, 伟 韩, 育红 陈, 于谦 孙, 昱 王, 菲菲 唐, 开彦 刘, 晓辉 张, 晓军 黄, 兰平 许

**Affiliations:** 1 北京大学人民医院、北京大学血液病研究所、国家血液系统疾病临床医学研究中心、造血干细胞移植北京市重点实验室，北京 100044 Peking University People's Hospital, Peking University Institute of Hematology, National Clinical Research Center for Hematologic Disease, Beijing Key Laboratory of Hematopoietic Stem Cell Transplantation, Beijing 100044, China; 2 郑州市第三人民医院血液科，郑州 450099 The Third People's Hospital of Zhengzhou, Zhengzhou 450099, China

**Keywords:** 兔抗人胸腺细胞免疫球蛋白, 不良反应, 预处理, 异基因造血干细胞移植, Antithymocyte globulin, Adverse reactions, Conditioning regimen, Allogeneic hematopoietic stem cell transplantation

## Abstract

**目的:**

探讨兔抗人胸腺细胞免疫球蛋白（rATG）第二次应用于异基因造血干细胞移植（allo-HSCT）的安全性。

**方法:**

回顾性分析2008年4月至2021年8月在北京大学血液病研究所先后接受两次allo-HSCT且预处理方案均使用rATG的27例患者。观察自rATG应用开始10 d内（移植前5 d至移植后3 d）预处理相关不良反应（发热、腹泻、心律失常、血压下降、肝损伤、癫痫等）在首次、二次应用中的发生情况，评价rATG在二次allo-HSCT中的安全性。

**结果:**

首次、二次allo-HSCT预处理期间自开始使用rATG至移植后3 d，不良反应发生率分别为96.3％、77.8％（*P*＝0.043），发热发生率分别为81.5％、63.0％（*P*＝0.129），腹泻发生率分别为59.3％、25.9％（*P*＝0.013），肝功能异常发生率分别为22.2％、25.9％（*P*＝0.750），其他事件（心律失常、血压下降、癫痫）的发生率分别为3.7％、18.5％（*P*＝0.083）。两次rATG应用期间发生的不良反应经对症治疗后均好转，无治疗中断发生。此外，在首次移植和二次移植应用rATG当天患者淋巴细胞计数的均数分别为0.5×10^9^/L、0.3×10^9^/L（*P*＝0.038）。

**结论:**

rATG用于第二次allo-HSCT没有增加预处理早期不良反应的发生率。

## Abstract (en)

**Objective:**

To look into the security of a second allogeneic hematopoietic stem cell transplantation（allo-HSCT）using rabbit anti-human thymocyte immunoglobulin（rATG）.

**Methods:**

Twenty-seven patients who used rATG in the first and second allo-HSCT at the Institute of Hematology, Peking University were enrolled in the study. Experienced toxicities associated with the conditioning protocol within 10 days（−5 d to +3 d）following the beginning of the rATG application, including fever, diarrhea, arrhythmia, reduced blood pressure, liver damage, seizures, and other problems.

**Results:**

The overall incidence of conditioning regimen early adverse reactions during the first transplantation and the second allo-HSCT conditioning regimen was 96.3％ and 77.8％（*P*＝0.043）. Fever rates were 81.5％ and 63.0％（*P*＝0.129）, diarrhea rates were 59.3％ and 25.9％（*P*＝0.013）, liver damage rates were 22.2％ and 25.9％（*P*＝0.75）, and the rates of other events（cardiac arrhythmia, low blood pressure, and epilepsy）were 3.7％ and 18.5％（*P*＝0.083）. Adverse reactions that occurred during both the first and second course of rATG applications have been improved with symptomatic treatment, and no treatment interruptions occurred.

**Conclusion:**

Reusing rATG in a second transplant was risk-free and did not result in higher early toxicities.

异基因造血干细胞移植（allo-HSCT）是治疗血液病的重要方法[1]，移植后疾病复发和植入失败显著影响移植患者的生存，二次allo-HSCT是挽救性治疗的重要措施之一[1]–[2]。抗胸腺细胞球蛋白（antithymocyte globulin, ATG）是“北京方案”单倍体造血干细胞移植（haplo-HSCT）的基石，具有预防GVHD和诱导免疫耐受的作用。有文献报道在重型再生障碍性贫血（SAA）患者免疫抑制（IST）治疗过程中，同类型ATG重复使用可能导致更严重的过敏、血清病等不良反应[3]–[4]。如果首次移植应用了ATG，二次移植采用同类型ATG是否会发生更重的不良反应，尚无报道。我们对两次移植预处理方案均使用兔抗人胸腺细胞免疫球蛋白（rATG）的血液病患者进行回顾性分析，观察预处理期间rATG相关早期不良反应发生情况，探讨二次移植预处理方案应用rATG的安全性。

## 病例与方法

一、病例

收集2008年4月28日至2021年8月21日在北京大学血液病研究所先后接受两次allo-HSCT且两次移植预处理方案中均使用rATG的患者，观察自rATG应用开始10 d内（−5～+3 d）预处理相关不良反应发生情况。

二、预处理方案

1. mBU/Cy+rATG方案：阿糖胞苷（Ara-C）4 g/m^2^，分2次静脉滴注，−9 d；白消安（Bu）0.8 mg/kg，每6 h 1次静脉滴注，−8～−6 d；环磷酰胺（Cy）1.8 g·m^−2^·d^−1^，静脉滴注，−5 d、−4 d；司莫司汀（MeCCNU）250 mg/kg，口服，−3 d；rATG 2.5 mg·kg^−1^·d^−1^，静脉滴注，−5～−2 d。用于白血病/骨髓增生异常综合征（MDS）患者。

2. Bu/Cy+rATG方案：Bu 0.8 mg/kg，每6 h 1次，静脉滴注，−7～−6 d；Cy 50 mg·kg^−1^·d^−1^，静脉滴注，−5～−2 d；rATG 2.5 mg·kg^−1^·d^−1^，静脉滴注，−5～−2 d。用于SAA患者。

3. TBI/Cy+rATG方案：TBI 770 cGy，−6 d；Cy 1.8 g·m^−2^·d^−1^，静脉滴注，−5 d、−4 d；MeCCNU口服，250 mg/m^2^，−3 d；rATG 2.5 mg·kg^−1^·d^−1^，静脉滴注，−5～−2 d。

三、预处理相关不良反应观察指标及处理

预处理相关不良反应分级依据《美国卫生及公共服务部常不良事件评价标准》（CTCAE）5.0分为1、2、3、4、5级。观察时间为rATG治疗后1～10 d（移植前5 d～移植后3 d）。主要观察指标：应用rATG期间所出现的不良反应：发热、腹泻、皮疹、转氨酶、胆红素、心律失常、黏膜损伤等指标。若rATG期间出现发热，留取血培养，加强抗生素及糖皮质激素应用；若出现腹泻，留取大便涂片和培养，给予蒙脱石散对症，并加强糖皮质激素应用。

四、统计学处理

计量资料的组间比较应用*t*检验，发生率应用卡方检验的组间比较，*P*<0.05为差异具有统计学意义。应用SPSS 22.0统计软件进行分析。

## 结果

一、一般情况

共收集病例27例，急性髓系白血病（AML）13例，急性淋巴细胞白血病（ALL）7例，MDS 3例，慢性髓性白血病（CML）2例，SAA 1例，慢性粒-单核细胞白血病（CMML）1例。首次allo-HSCT中，24例为haplo-HSCT，3例为无关供者全相合造血干细胞移植（MUD-HSCT），预处理方案均为mBU/Cy+rATG方案。在二次allo-HSCT中，haplo-HSCT 25例，MUD-HSCT 2例，24例采用TBI/Cy+rATG预处理方案，3例采用mBU/Cy+rATG预处理方案。两次移植中位间隔时间为15.4（6.6～107.4）个月。全部27例患者疾病资料见表1。

**表1:** 27例二次异基因造血干细胞移植血液病患者临床特征及转归

例号	性别	年龄（岁）	原发病	首次移植类型	两次移植间隔（月）	二次移植前疾病状态	二次移植类型	替换供者	二次移植预处理方案	随访时间（月）	转归
1	女	28	MDS-EB2	HID	39.2	NR	HID	否	TBI/Cy	4.5	MRD（+）
2	男	20	AML	HID	8.4	NR	HID	否	TBI/Cy	5.5	MRD（+）
3	男	47	AML	MUD	27.3	NR	HID	是	mBU/Cy	3.2	MRD（+）
4	男	16	T-ALL	HID	11.4	MRD（+）	HID	否	mBU/Cy	22.1	MRD（+）
5	女	20	CML	HID	23.3	NR	HID	否	TBI/Cy	24.3	MRD（+）
6	男	16	CML	HID	11.2	NR	HID	否	TBI/Cy	–	NR
7	女	45	AML	HID	21.8	MRD（+）	HID	否	TBI/Cy	11.2	MRD（+）
8	女	37	MDS-EB2	HID	8.2	NR	HID	否	TBI/Cy	2.9	MRD（+）
9	男	6	AML	HID	6.9	NR	HID	否	TBI/Cy	–	NR
10	男	36	AML	MUD	26.9	MRD（+）	MUD	是	TBI/Cy	8.2	LFS
11	女	26	B-ALL	HID	11.4	MRD（+）	HID	是	TBI/Cy	3.9	LFS
12	男	32	B-ALL	HID	36.3	CR	HID	是	TBI/Cy	15.1	LFS
13	女	24	AML	HID	49.1	MRD（+）	HID	否	TBI/Cy	34.3	LFS
14	男	31	AML	HID	20.7	MRD（+）	HID	是	TBI/Cy	5.6	MRD（+）
15	女	35	MDS-EB1	HID	15.4	MRD（+）	HID	否	TBI/Cy	2.8	TRM
16	女	11	SAA	MUD	9.4	GF	HID	是	mBU/Cy	40.2	LFS
17	男	27	B-ALL	HID	30.0	MRD（+）	HID	是	TBI/Cy	9.6	MRD（+）
18	女	19	AML	HID	8.2	NR	HID	否	TBI/Cy	2.1	MRD（+）
19	女	26	AML	MUD	90.5	NR	HID	是	TBI/Cy	1.6	MRD（+）
20	男	27	B-ALL	HID	9.4	CR	HID	是	TBI/Cy	19.4	LFS
21	男	48	AML	HID	9.8	MRD（+）	HID	是	TBI/Cy	18.0	LFS
22	男	19	AML	HID	10.4	MRD（+）	HID	是	TBI/Cy	2.4	MRD（+）
23	男	41	T-ALL	HID	79.9	MRD（+）	HID	是	TBI/Cy	13.3	LFS
24	女	34	AML	HID	32.9	CR	HID	是	TBI/Cy	18.1	LFS
25	男	60	AML	HID	9.6	NR	HID	是	TBI/Cy	0.4	TRM
26	男	35	CMML	HID	6.6	NR	HID	是	TBI/Cy	3.8	MRD（+）
27	男	32	T-ALL	MUD	107.4	MRD（+）	MUD	是	TBI/Cy	6.5	MRD（+）

注：AML：急性髓系白血病；B-ALL、T-ALL分别为急性B淋巴细胞白血病、急性T淋巴细胞白血病；MDS-EB：骨髓增生异常综合征伴原始细胞增多；SAA：重型再生障碍性贫血；CML：慢性髓性白血病；CMML：慢性粒-单核细胞型白血病；HID：单倍体造血干细胞移植；MUD：无关供者全相合造血干细胞移植；TBI：全身放射治疗；Cy：环磷酰胺；LFS：无白血病生存；MRD（+）：微小残留病阳性；CR：完全缓解；NR：未缓解；GF：植入失败；TRM：移植相关死亡；随访时间：MRD（+）患者为事件发生时间，LFS患者为末次复查时间

二、预处理早期不良反应

1. 非感染性发热：首次allo-HSCT应用rATG期间22例（81.5％）患者出现发热（1级13例，2级9例），中位最高体温38.5（37.5～40.2）°C。二次allo-HSCT应用rATG期间17例（63.0％）患者出现发热（1级10例，2级6例，3级1例），中位最高体温38.7（37.5～40.8）°C。两组患者发热发生率差异无统计学意义（*P*＝0.129）。所有发热患者血培养结果均为阴性。

2. 非感染性腹泻：首次allo-HSCT应用rATG期间16例（59.3％）患者发生腹泻（1级3例，2级9例，3级4例），中位腹泻量450（225～2 300）ml。二次allo-HSCT应用rATG期间7例（25.9％）患者发生腹泻（1级1例，2级5例，3级1例），中位腹泻量480（365～1 050）ml。两组患者腹泻发生率差异有统计学意义（*P*＝0.013）。

3. 肝损伤：首次allo-HSCT应用rATG期间6例（22.2％）患者发生肝损伤（1级2例，2级2例，3级1例，4级1例）。二次allo-HSCT应用rATG期间7例（29.6％）患者发生肝损伤（1级6例，2级1例）。两组患者肝损伤发生率差异无统计学意义（*P*＝0.750）。

4. 其他并发症：首次allo-HSCT应用rATG期间合并心律失常1例（−4 d，阵发性室上性心动过速），无血压下降及癫痫发作患者。二次allo-HSCT应用rATG期间合并心律失常2例（1例−3 d发生窦性心动过缓，1例+2 d发生阵发性室上性心动过速）、血压下降2例（分别发生于−5 d、−4 d），癫痫发作1例（+2 d）。两组患者其他并发症的发生率分别为3.7％、18.5％（*P*＝0.083）。

首次、二次allo-HSCT预处理期间早期不良反应总发生率分别为96.3％、77.8％（*P*＝0.043）。全部27例患者两次allo-HSCT应用rATG期间对于预处理毒性均耐受良好，未发生治疗中断。

三、首剂rATG应用当天淋巴细胞计数

淋巴细胞作为rATG最主要的靶细胞，可能与rATG引起的不良反应相关，但是由于患者疾病状态、预处理方案等不同导致应用rATG时淋巴细胞计数不同。我们统计了24例二次移植更换为TBI/Cy方案的患者在两次预处理首次应用rATG当天淋巴细胞计数情况，均数分别为0.5×10^9^/L、0.3×10^9^/L（*P*＝0.038），如图1。

**图1:**
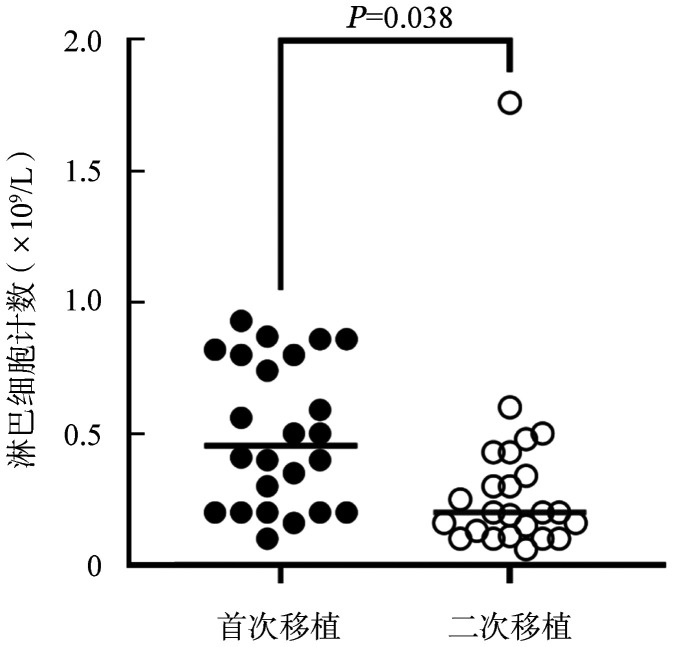
两次allo-HSCT应用兔抗人胸腺细胞免疫球蛋白（rATG）当天外周血淋巴细胞计数的比较

## 讨论

allo-HSCT为血液病患者带来了治愈的希望，但约有四分之一的白血病患者在首次allo-HSCT后复发，严重影响患者的生存[5]。二次allo-HSCT具有较强的移植物抗白血病（GVL）作用，换用新的单倍体供者可以一定程度上避免了移植时的HLA染色体区域的染色体杂合性缺失（HLA-loss），增强GVL效应，使部分复发患者再次获得长期无病生存[6]–[9]。然而二次allo-HSCT比首次allo-HSCT更加复杂，移植相关死亡一直是困扰临床的难题。本研究发现rATG应用于二次移植，预处理期间的不良反应并无增加，说明rATG重复应用是安全的。

rATG可并发多种输注反应，如发热、寒战、呼吸困难、皮疹、腹泻、肝细胞破坏、心律失常等，甚至过敏性休克，大多数症状可归因于细胞因子释放综合征（CRS），而且是可逆的。血清病的反应多发生于开始用药的5～15 d[10]，由于感染、植入综合征、急性GVHD等并发症，其临床表现可能与预处理早期毒性重叠，因此我们对rATG安全性观察时间截止于移植后早期。

刘代红等[11]曾对采用mBu/Cy方案及mBu/Cy+ATG方案进行预处理的allo-HSCT患者在预处理期间及移植后早期发生的不良反应进行了对比，两组发热发生率分别为4.8％、81.0％，腹泻发生率分别为59.5％和79.3％，胆红素升高发生率分别为16.7％、48.3％，提示含rATG的预处理方案发热、腹泻、肝损害的发生率明显高于不含rATG的预处理方案。本组27例allo-HSCT患者二次移植与首次移植预处理期间早期总体不良反应发生率分别为77.8％、96.3％（*P*＝0.043），其中最常见的不良反应是发热、腹泻、肝损伤，其中首次移植组腹泻发生率较高（59.3％对25.9％，*P*＝0.013）。周洁等[12]对比了mBu/Cy方案（20例）和TBI/Cy方案（159例）的优缺点，mBu/Cy方案组的肝损害发生率明显高于TBI组（80％对48％，*P*<0.05），出血性膀胱炎发生率分别为50％、19％（*P*<0.05），其他不良反应（胃肠道反应、口腔黏膜炎、间质性肺炎）无明显差异，与我们所观察的含有rATG方案的预处理早期常见不良反应有所不同，说明rATG应用期间出现的发热、腹泻等常见不良反应与rATG密切相关。

值得注意的是，rATG应用期间心脏、中枢神经系统等重要脏器不良反应发生率在二次移植中有增高趋势（3.7％对18.5％，*P*＝0.083）。在首次移植中，1例患者于−5 d发生阵发性室上性心动过速，考虑与Cy相关，经暂停Cy、应用艾司洛尔控制心室率治疗后好转。在二次移植中，有另外两例患者出现心脏相关不良反应，1例于+2 d发生阵发性室上性心动过速伴ST段抬高，考虑为Cy所致心肌炎，经美托洛尔、加用肾上腺糖皮质激素治疗后恢复；1例于−4 d发生心动过缓，考虑为异丙嗪药物不良反应，经暂停异丙嗪后心率恢复。二次移植中有1例患者+2 d发生癫痫大发作，最终诊断为可逆性后部脑病综合征，经镇静、降颅压治疗后好转。二次移植中血压下降2例，均伴有39 °C以上体温，考虑与rATG相关，经减慢rATG输注、加强肾上腺糖皮质激素及补液治疗后血压稳定，体温正常。两组均无因rATG相关毒性停止或改变预处理方案病例。Gupta等[3]关于18例SAA患者第三次应用ATG治疗的临床研究中，有2例患者分别在ATG给药的第2天和第3天出现高热、血压下降和皮疹，经糖皮质激素和抗组胺药治疗后不能纠正，在停止ATG治疗后这些症状迅速消失，并且回顾了该中心在过去的11年中由于ATG相关不良反应停药的患者，第1个疗程停药发生率<1％（1/122），第2个疗程约4％（2/49），第三个疗程约15％（2/13），显示严重不良反应的发生率呈上升趋势。虽然第二次allo-HSCT再次应用包含ATG的预处理方案耐受性良好，但是需密切监测患者的生命体征，警惕严重不良反应的发生。

ATG引起的CRS和其他形式的输注反应是无法通过皮试预测的，但是ATG结合在靶细胞（淋巴细胞、单核细胞、树突状细胞）表面导致细胞因子产生和系统性炎症反应，首剂输注时淋巴细胞数量越高，越容易发生CRS[13]。减低强度预处理（RIC）方案被报道与更严重的CRS相关，因为与清髓性预处理（MAC）方案相比RIC方案中可能有更多的残余淋巴细胞[14]。Soiffer等[15]发现TBI方案较BU方案具有更强的清除淋巴细胞作用。我们观察的24例二次移植应用TBI/Cy+rATG方案患者在应用rATG期间，首次移植总体不良反应高于二次移植，可能与首次移植中开始应用rATG当天淋巴细胞计数高于二次移植组（*P*＝0.038）有关。

本研究结果显示，rATG用于第二次allo-HSCT 没有增加预处理早期不良反应的发生率。ATG的多次应用需警惕严重不良反应的发生。淋巴细胞是ATG药代动力学的唯一相关预测因子[16]，或许基于ATG用药时的淋巴细胞绝对计数而不仅仅是患者体重来计算ATG的用药更精准。不同预处理方案中ATG剂量是否相同，仍需进一步探索。
